# Flint Hills Phages: Isolation Parameters and Genomic Characteristics of 23 Phages

**DOI:** 10.17912/micropub.biology.001461

**Published:** 2025-05-28

**Authors:** Martha Smith-Caldas, Christopher Herren, Victoria Thurm, Dasik Clouse, Aidan Ozga, Elizabeth Herrman, Ashley Hine, Kyla Hornback, Nathan Jones, Erika Kline, Lexi Pitts, Rece Buckmaster, Camille Carrier, Marie Dios, MacKenzie Dunigan, Selah Hageman, Maddy Kang, Molly Kang, Madison Sims, Landon Taylor, Bailey Wilson, Taz Zeigler

**Affiliations:** 1 Biology, Kansas State University, Manhattan, Kansas, United States

## Abstract

Bacteriophages reported in this announcement were isolated on *Mycobacterium smegmatis* mc ^2^ 155, *Microbacterium foliorum* NRRL B-24224, and *Gordonia terrae* CAG3. The 24 phages span 19 different clusters, and range in genome length from 41.8 kbp to 151.1 kbp. Phage CherryTomatoes is only the fourth reported actinobacteriophage isolated using *G. terrae* that possesses a myovirus morphology.

**Table 1. Twenty-four bacteriophages isolated from soil samples collected in the Flint Hills region of Kansas:** GPS, host, cluster identification, genomic characteristics, and GenBank accession numbers for 24 isolated bacteriophages.

**Table:** 

Phage Name; Host Species	Sample GPS Coordinates	Isolation Method	No. of 150-base Reads; Coverage (fold)	Genome Length (bp); GC Content (%)	No. of Putative Genes; No. of tRNAs	Cluster	Genome Ends Characteristics	Sequence Read Archive Accession	GenBank Accession.
ACFishhook; M. smegmatis mc^2 155	39.101453 N, 96.61059 W	Enriched	789,139; 2334	47343; 64.0%	77; 3	A3	3' single stranded overhang: 5'-CGGGTGGTAA	SRX26413415	MK284518
Arti; G. terrae CAG3	39.244623 N, 96.317539 W	Enriched	522,145; 1111	66539; 66.0%	91; 0	CR2	3' single stranded overhang: 5'-CGCCGCGTAC	SRX26413416	OR434020
BenoitCattle; G. terrae CAG3	39.78215 N, 98.40191 W	Direct	479,471; 887	77049; 58.6%	109; 1	CS2	Direct Terminal Repeat	SRX26413427	PQ184834
BiggityBass; G. terrae CAG3	39.108056 N, 96.596944 W	Direct	411,782; 944	63202; 69.4%	83; 1	DR	Circularly Permuted	SRX1602227	ON260813
BubbaBear; M. foliorum NRRL B-24224	39.195682 N, 96.575696 W	Enriched	57,689; 58	41814; 66.6%	68; 1	EB	3' single stranded overhang: 5'-TCTCCCGGCA	SRX26413432	MK814753
Buttrmlkdreams; G. terrae CAG3	39.1934 N, 96.5965 W	Enriched	171,607; 526	45999; 60.4%	71; 0	CT	3' single stranded overhang: 5'-CGGTAGGCTT	SRX26413433	MT776809
ChadMasterC; G. terrae CAG3	38.821389 N, 94.751944 W	Enriched	3.8M; 9199	59685; 67.7%	84; 0	DE1	Circularly Permuted	SRX26413434	ON081332
CherryTomatoes; G. terrae CAG3	37.62855 N, 97.48207 W	Enriched	238,293; 224	151645; 66.0%	236; 1	DO	Circularly Permuted	SRX26413435	PP978796
Chill; M. smegmatis mc^2 155	39.1951 N, 96.58535 W	Enriched	237,119; 506	64529; 59.7%	89; 0	D1	Circularly Permuted	SRX26413436	MK524498
Crater; G. terrae CAG3	39.186 N, 96.571 W	Enriched	967,428; 2681	52539; 63.0%	100; 0	DN3	3' single stranded overhang: 5'-CGTTAGGCAT	SRX26413437	OR434025
DBQu4n; M. smegmatis mc^2 155	39.2445 N, 96.316861 W	Enriched	542,596; 1451	52724; 63.5%	93; 5	A2	3' single stranded overhang: 5'-CGGTCGGTTA	SRX26413438	MK494087
Durga; M. smegmatis mc^2 155	39.192363 N, 96.585999 W	Enriched	463,955; 966	68866; 66.4%	101; 0	B1	Circularly Permuted	SRX26413418	MK279850
EnalisNailo; G. terrae CAG3	39.18 N, 96.58 W	Enriched	350,122; 1015	51094; 67.1%	76; 0	CY1	3' single stranded overhang: 5'-CGTATGGCAT	SRX26413419	MK820641
Faith5x5; G. terrae CAG3	39.19232 N, 96.584034 W	Enriched	120,324; 408	41982; 65.1%	71; 0	CZ6	3' single stranded overhang: 5'-TCGTCGGGGTGA	SRX26413420	MN585966
Halo3; G. terrae CAG3	39.24492 N, 96.316794 W	Enriched	388,403; 938	59182; 67.9%	94; 0	DC1	Circularly Permuted	SRX26413421	OR521081
Hamood; G. terrae CAG3	39.19416 N, 95.58667 W	Enriched	645,021; 145	50919; 67.0%	76; 0	D1	3' single stranded overhang: 5'-TGCCGCGGTA	SRX26413422	PQ244015
MoonTowerMania; G. terrae CAG3	38.83372 N, 95.25346 W	Enriched	512,053; 1226	59932; 67.6%	87; 0	DE1	Circularly Permuted	SRX26413424	OR283206
PinkCoffee; G. terrae CAG3	38.83372 N, 95.25346 W	Enriched	846,945; 2071	58559; 67.8%	96; 0	DC1	Circularly Permuted	SRX26413425	MZ622177
Pollywog; M. smegmatis mc^2 155	39.192467 N, 96.58427 W	Enriched	685,602; 1658	58397; 61.3%	108; 0	F1	3' single stranded overhang: 5'-CCGATGGCAT	SRX26413426	MK359343
Rabbitrun; G. terrae CAG3	39.0458 N, 96.963 W	Enriched	339,204; 629	76821; 58.8%	125; 5	DU2	3' single stranded overhang: 5'-ATCTGCCTCAC	SRX26413428	MT658805
RiverRaider; G. terrae CAG3	39.7265 N, 97.7825 W	Direct	394,725; 990	56931; 67.6%	86; 0	DE1	Circularly Permuted	SRX26413429	PQ184823
WaldoWhy; M. smegmatis mc^2 155	39.045 N, 96.856 W	Enriched	335,516; 735	64529; 59.7%	89; 0	D1	Circularly Permuted	SRX26413430	MK494102
Wocket; G. terrae CAG3	39.182607 N, 96.583822 W	Enriched	280,356; 811	49767; 67.1%	79; 0	CV	3' single stranded overhang: 5'-TCGCCGGTGA	SRX26413431	MN585963

## Description

Bacteriophages are important key biological agents that regulate bacterial populations (Hatfull GF, 2015). The isolation and characterization of bacteriophages can advance our understanding of microbial population dynamics and the development of therapeutics for controlling bacterial growth (Hatfull GF, 2022). Here, we describe 24 bacteriophages isolated from soil samples collected in the Flint Hills region of Kansas (GPS coordinates provided in Table 1) using various actinobacteria as hosts. In total, 16 phages were isolated on *Gordonia terrae* CAG3, 6 phages on *Mycobacterium smegmatis* mc ^2^ 155, and 1 phage on *Microbacterium foliorum* NRRL B-24224.

All phages were isolated by washing the soil samples in liquid medium and filtering the wash using a 0.2 µm filter. The medium used to grow *G. terrae* CAG3 and *M. foliorum* NRRL B-24224 was PYCa, and the medium used to grow *M. smegmatis* mc ^2^ 155 was 7H9. The filtrate was then plated in top agar with host bacteria (direct isolation) or first inoculated with host bacteria and incubated with shaking at 30°C for 1 – 3 days before being refiltered and plated in top agar with host bacteria (enriched isolation) (Table 1). Plates were incubated for 1 – 3 days at 30˚C to form plaques, which were then plaque-purified through 2 – 3 rounds of additional plating. Liquid lysates were then prepared for each purified phage, which were used to image virions by negative-stain (1% uranyl acetate) transmission electron microscopy. All phages displayed a siphovirus morphology, with the exception of CherryTomatoes, which possesses a myovirus morphology. Phage DNA was extracted from the lysate using the Wizard DNA prep kit from Promega, and prepared for sequencing using the NEB FS Ultra II kit before being sequenced using an Illumina MiSeq (v3 reagents) to generate 150 base reads. Raw reads were then assembled using Newbler v2.9 and checked for completeness and genome ends using Consed v29 (Gordon et al., 1998). Sequencing parameters and genome characteristics are presented in Table 1.

All genomes were annotated using DNAMaster (http://cobamide2.bio.pitt.edu/, v5.0.2) and PECAAN (https://discover.kbrinsgd.org , v20240320), Glimmer v3.02 (Delcher et al. 2007, v3-3.02b), GeneMark v2.5 (Besemer and Borodovsky 2005), and Phamerator v578 (Cresawn et al. 2011, web version). tRNAs were identified using ARAGORN v1.2.41 (Laslett and Canback 2004) and tRNAscan-SE (Lowe and Eddy 1997). Functional assignment was performed using BLAST (Altschul et al. 1990.), searching against the Actinobacteriophage and NCBI non-redundant databases, and HHPRED (Soding et al. 2005), searching against the PDB_mmCIF70, Pfam- v.36, NCBI Conserved Domains databases. Phages were assigned to clusters based on gene content similarity of >35% to phages in the Actinobacteriophage database, phagesDB (https://phageDB.org) (Russell and Hatfull 2017; Pope et al. 2017). All software were used with default settings. Genome content is described in Table 1.

Each genome has a set of genes related to phage structure and assembly, including major capsid, minor tail, tape measure, terminase, and portal protein. These genes were located in the right arm of the genome, except for phage CherryTomatoes, where they are located in the middle of the genome. Among the phages characterized in this study, terminase is indicated as either a single gene or two genes (small and large subunits).
